# Therapeutic Benefits of Short-Arm Human Centrifugation in Multiple Sclerosis–A New Approach

**DOI:** 10.3389/fneur.2021.746832

**Published:** 2022-01-04

**Authors:** Chrysoula Kourtidou-Papadeli, Christos A. Frantzidis, Christos Bakirtzis, Anatoli Petridou, Sotiria Gilou, Aliki Karkala, Ilias Machairas, Nikolaos Kantouris, Christiane M. Nday, Emmanouil V. Dermitzakis, Eleftherios Bakas, Vassilis Mougios, Panagiotis D. Bamidis, Joan Vernikos

**Affiliations:** ^1^Biomedical Engineering and Aerospace Neuroscience (BEAN), Laboratory of Medical Physics and Digital Innovation, School of Medicine, Aristotle University of Thessaloniki, Thessaloniki, Greece; ^2^Greek Aerospace Medical Association and Space Research (GASMA-SR), Thessaloniki, Greece; ^3^Laboratory of Aerospace and Rehabilitation Applications “Joan Vernikos”, AROGI Rehabilitation Centre, Thessaloniki, Greece; ^4^Aeromedical Center of Thessaloniki (AeMC), Thessaloniki, Greece; ^5^Department of Neurology, Multiple Sclerosis Center, AHEPA University Hospital, Aristotle University of Thessaloniki, Thessaloniki, Greece; ^6^Laboratory of Evaluation of Human Biological Performance, School of Physical Education and Sport Science at Thessaloniki, Aristotle University of Thessaloniki, Thessaloniki, Greece; ^7^Thirdage LLC, Culpeper, VA, United States

**Keywords:** artificial gravity, brain plasticity, case report, expanded disability status scale, muscle oxygenation, secondary progressive multiple sclerosis

## Abstract

Short-arm human centrifugation (SAHC) is proposed as a robust countermeasure to treat deconditioning and prevent progressive disability in a case of secondary progressive multiple sclerosis. Based on long-term physiological knowledge derived from space medicine and missions, artificial gravity training seems to be a promising physical rehabilitation approach toward the prevention of musculoskeletal decrement due to confinement and inactivity. So, the present study proposes a novel infrastructure based on SAHC to investigate the hypothesis that artificial gravity ameliorates the degree of disability. The patient was submitted to a 4-week training programme including three weekly sessions of 30 min of intermittent centrifugation at 1.5–2 *g*. During sessions, cardiovascular, muscle oxygen saturation (SmO_2_) and electroencephalographic (EEG) responses were monitored, whereas neurological and physical performance tests were carried out before and after the intervention. Cardiovascular parameters improved in a way reminiscent of adaptations to aerobic exercise. SmO_2_ decreased during sessions concomitant with increased *g* load, and, as training progressed, SmO_2_ of the suffering limb dropped, both effects suggesting increased oxygen use, similar to that seen during hard exercise. EEG showed increased slow and decreased fast brain waves, with brain reorganization/plasticity evidenced through functional connectivity alterations. Multiple-sclerosis-related disability and balance capacity also improved. Overall, this study provides novel evidence supporting SAHC as a promising therapeutic strategy in multiple sclerosis, based on mechanical loading, thereby setting the basis for future randomized controlled trials.

## Introduction

Multiple sclerosis (MS) is a multifactorial and often disabling disease of the central nervous system, frequently with unpredictable progression, due to inflammation-induced demyelination and axonal damage (1), leading to inactivity, deconditioning and physical disability (2). To date, proposed relapse-preventing medications have immunosuppressive and immunomodulatory effects that target the lymphocyte number, proliferation and trafficking, as well as cytokine production. By acting on the immune system that is dysregulated in multiple sclerosis, those medications partially control central nervous system inflammation, preventing the occurrence of relapses and new inflammatory lesions. They focus on slowing the disease progression and alleviating the symptoms, thus steering the researchers' attention toward neuroprotective and repair-promoting strategies (3) like exercise (4). Recommendations for exercise and lifestyle physical activity were recently made to attenuate the MS-related progression of physical disability by improving physiological function and optimal functional brain reorganization (5, 6). Alternative exercise training strategies, capable of better managing physical disability, emerge as a need.

The medications used to treat MS have immunosuppressive and immunomodulatory effects that target the lymphocyte number, proliferation and trafficking, as well as cytokine production.

Research on deconditioning due to lack of gravity in microgravity environment and deconditioning due to neurodegenerative diseases has shown common effects on muscle strength and function, as well as on the cardiovascular and vestibular systems. Immobility and lack of exercise induce muscle atrophy and bone demineralization, playing thus a primary role in the accumulation of disability (6). Whereas the primary physiological response to spaceflight derives from lack of stimulus to the gravity sensors, the primary physiological response to neurodegenerative diseases derives from lack of exposure to gravity due to disability. The scientific community recognized the effects of mechanical loading on tissue regeneration (7) through alterations in the expression of genes that permit axonal sprouting (8), suggesting that the combination of increasing gravitational load with movement therapy on a centrifuge in individuals with physical disabilities would be more effective than movement therapy alone (9).

Thus, our hypothesis is that the application of increased gravitational stimuli intermittently using short-arm human centrifugation (SAHC) produces biological responses mimicking those of whole-body exercise (10), which may serve as an innovative therapeutic approach against deconditioning (11). The beneficial effect of artificial gravity is tested through a series of outcome measures, estimating its impact on cardiovascular parameters such as cardiac output, stroke volume and heart rate as well as neurophysiological parameters (cortical oscillatory activity and functional connectivity). In addition, muscle oxygenation, neurological, mobility and balance tests were used to provide an estimate of changes in muscle function and sensorimotor coordination (10). We anticipated increased activation and functional connectivity alterations within neuroplasticity associated brain regions (11) and enhancement of cortical excitability (12) due to the SAHC intervention. Based on the outcomes of the present case study, we propose a rehabilitation intervention strategy on a SAHC device suitable to counter deconditioning, which may play a crucial role in changing the course of the disease. Furthermore, it might provide a new therapeutic approach for individuals with physical disabilities and the elderly, providing yet another terrestrial application of space research.

### Patient Report

To investigate our hypothesis, a 54-year-old Caucasian male with secondary progressive multiple sclerosis (SPMS) was enrolled in this study. The participant's height is 1.70 m and his weight was 80 kg (BMI: 27.682). Disease onset was at the age of 20, with an episode of numbness in the left extremities, which resolved fully after a few days without any medical intervention. MS was diagnosed at the age of 26, during his hospitalization due to left optic neuritis. At that time, brain MRI revealed periventricular, subcortical and infratentorial demyelinating lesions, while additional lesions were observed in the cervical cord. Lumbar puncture revealed the presence of specific oligoclonal bands in the central nervous system (type 2). During the relapsing-remitting disease phase, he was treated with interferons, followed by natalizumab. Treatment with disease-modifying drugs was discontinued due to the transition to SPMS (gradual decline of walking ability, attributable to progressive spastic ataxic paraparesis), 3 years before the intervention presented herein. At screening, he was under symptomatic treatment with baclofen, fampridine and duloxetine. No changes regarding treatment were made during the 4-week study period. Besides SPMS, his medical and family history was unremarkable.

Neurological examination revealed significant decrease in visual acuity in the left eye (20/100) with a large central scotoma, mild impairment of the superficial sense in the right upper extremity, mild decrement of vibration sense in both lower extremities, spastic paraparesis (Medical Research Council muscle-grading scale 3/5 in the left leg, 4/5 in the right leg), with extensor plantar responses in both lower extremities, non-sustained clonus in the right leg and sustained clonus in the left leg. The patient used intermittent self-catheterization while presenting spastic ataxic gait with unilateral assistance (Expanded Disability Status Score 6.0). Although a recent MRI was not available, he had presented no relapses of the disease during the last 3 years, while previous MRIs did not present any signs of inflammatory activity. Thus, he was classified as having non-active SPMS according to the criteria of Lublin et al. (13).

## Methods

### Ethics

The patient was informed about the aims of the rehabilitation programme and the upcoming measurements, and he was given the opportunity to ask any questions he had regarding his involvement. Then he signed a written informed-consent form, which was previously approved by the Bioethics Committee of the School of Medicine of the Aristotle University of Thessaloniki (179/19.03.2020). The therapeutic intervention was registered in the Clinical Trials Repository (NCT04369976).

### Experimental Design

**Figure 1:**
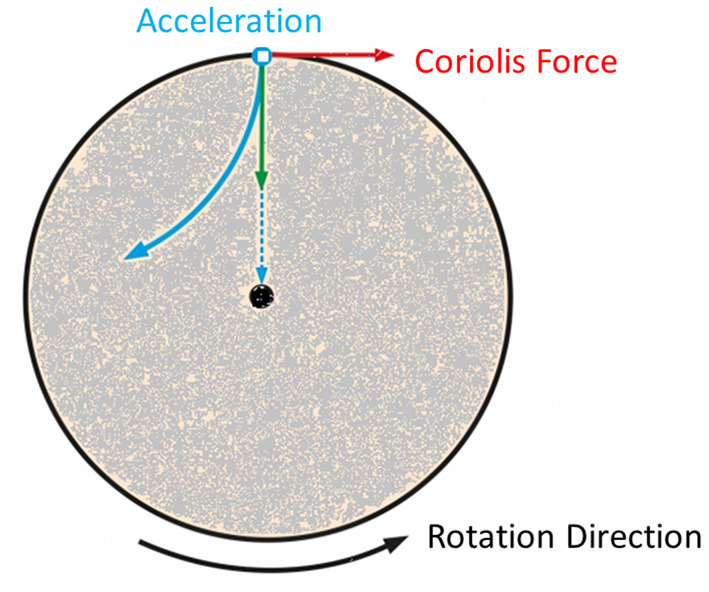
Visualization of the forces induced due to centrifugation and more specifically the Coriolis force.

The patient was submitted to a 10-min centrifugation trial at 1 g for familiarization and was enrolled in the study, since he tolerated the procedure without dizziness or nausea. He was advised to abstain from caffeine during training days, avoid alcohol and any medications during the 12 h preceding the training session, avoid eating during the preceding 2 h and avoid heavy exercise during the preceding 24 h. When artificial gravity is produced by centrifugation as seen in Figure 1, the rotating environment gives rise to cross-coupled Coriolis force, acting in a direction that is perpendicular both to the direction of the velocity of the moving subject mass and to the axis of rotation, which is known to induce nystagmus and discomfort. To mitigate the Coriolis side effect, the participant was strapped to a centrifuge of a 2-m radius with safety harnesses, and the participant's head was placed stable near the center of the centrifuge.

**Figure 2:**
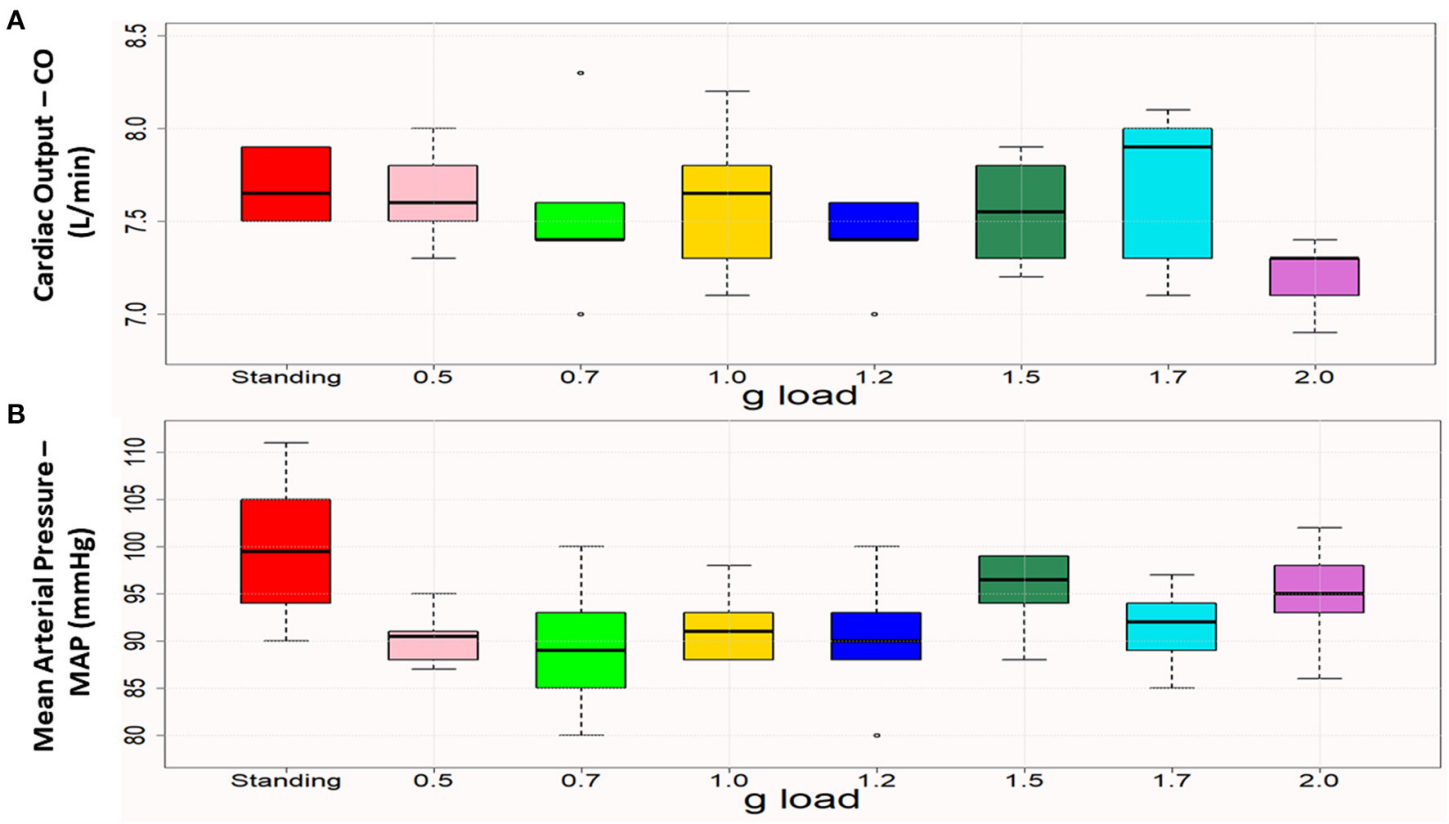
Boxplots of cardiac output **(A)** and mean arterial pressure **(B)** in standing and the various (0.5–2.0) g loads. Each box represents the interquartile range, and the centerline represents the median. Whiskers are extended to the most extreme data point that is no more than 1.5 times the interquartile range from the edge of the box (Tukey style). Dots represent outliers. Cardiac output was close to the standing values at 1 g and 1.5 g, and mean arterial pressure was closest to the standing value at 1.5 g.

The patient was submitted to an initial centrifugation test with gradually increasing gravitational loads to let us select the optimal load for the 4-week training programme, based on the cardiac output (CO) and mean arterial pressure (MAP) of the patient while standing. The g-load that leads the individual close to those values is considered optimal for training. These data for a healthy, young cohort were analyzed in a previous study of our group (14), while the patient-specific parameters are visualized in Figure 2. The test started at 0.5 *g* and was followed by 0.7, 1.0, 1.2, 1.5, 1.7, and 2 g, each for 5 min, with gradual acceleration and deceleration and with 6 min of pause between *g* loads. According to this initial assessment, it was decided that each training session would consist of a total of 30 min of centrifugation at 1.5, 1.7, 1.7, 1.7, 2, and 2 g, each for 5 min, with 6 min of pause in between (Figure 2). The total time of the session (including preparatory and recovery steps) was ~1 h and a half, and the training frequency was three times per week.

During sessions, cardiovascular, muscle oxygen saturation (SmO_2_) and electroencephalographic (EEG) responses were monitored. Before and after the 4-week training programme, neurological and physical performance tests were carried out. Additionally, before and after the 1st centrifugation session, balance was assessed.

### SAHC Rehabilitation Infrastructure

#### Theoretical Concept

SAHC is an integrated multi-system countermeasure in order to provide artificial gravity training for rehabilitation purposes. It targets physiological deconditioning imposed by inactivity or lack of gravitational force. The AG training effects on either humans or animals are equivalent to those of natural gravity. It functions by exerting a centrifugal force on a body accelerated centripetally in a rotating device (15–17). The participant lies in a supine and horizontal position on the rotation bed with the head toward the center. Patients are forced away from the axis of rotation with a force that is the product of body mass, distance from the axis of rotation and angular velocity squared. This force can be calculated from the equation F = mrw^2^, where m is the mass of the subject, r is the distance from the center of the centrifuge to the center of mass of the subject and w is the velocity at which the bed rotates. Thus, force exerted at the feet increases with the velocity of rotation and with the body's distance from the axis of rotation. Because the head is placed close to the axis, the *g* load at the head is close to zero, thus preventing a Coriolis side effect, while the *g* load at the feet may reach 3.5 g at the maximal velocity of the device.

#### The Physiological Impact of Artificial Gravity

The SAHC creates a centrifugal force which mimics gravity. Centrifugal forces are sensed by sensory receptors (transducers housed in sense organs) which are recognized as regulators of cell structure and function. These forces transduce the mechanical properties of cells into chemical signals, translating the external energy to internal electrical impulses, encoding thus the information from the external environment. Stretch, compression and fluid shifts are transduced through intra and intercellular metabolic signals to vascular, neural and other networks to affect a response (18). Let us regard the example of the osteoblasts, which have been shown to respond to the mechanical stress. Both the type of stress and its degree determine the osteoblast response with possible regulatory sensors to include mechano-sensitive calcium channels or other receptors (cytokine, GF growth factor related kinases).

AG training aims to counter deconditioning through the simultaneous activation of gravity receptors and hydrostatic pressure. Both mechanisms are activated during the linear centripetal acceleration. Since gravitational force acts on cell mass increasing cell weight, gravity variations have direct effects on cell/substrate interactions (e.g., adhesion), cytoskeletal conformation, activation of stretch-activated receptors, transduction pathways and gene expressions. Indirect effect such as those mediated by the hydrostatic pressure and fluid shear flow also take place (19). Simultaneously, AG-induced acceleration causes both increased load on muscles and bones and bone remodeling. The load increments are similar with Earth's gravity leading to subsequent blood flow increases regarding the lower body muscles (20). Bone remodeling involves the functioning of osteocytes as strain receptors due to the disposition of interstitial fluid (21).

#### The Proposed Infrastructure

**Figure 3:**
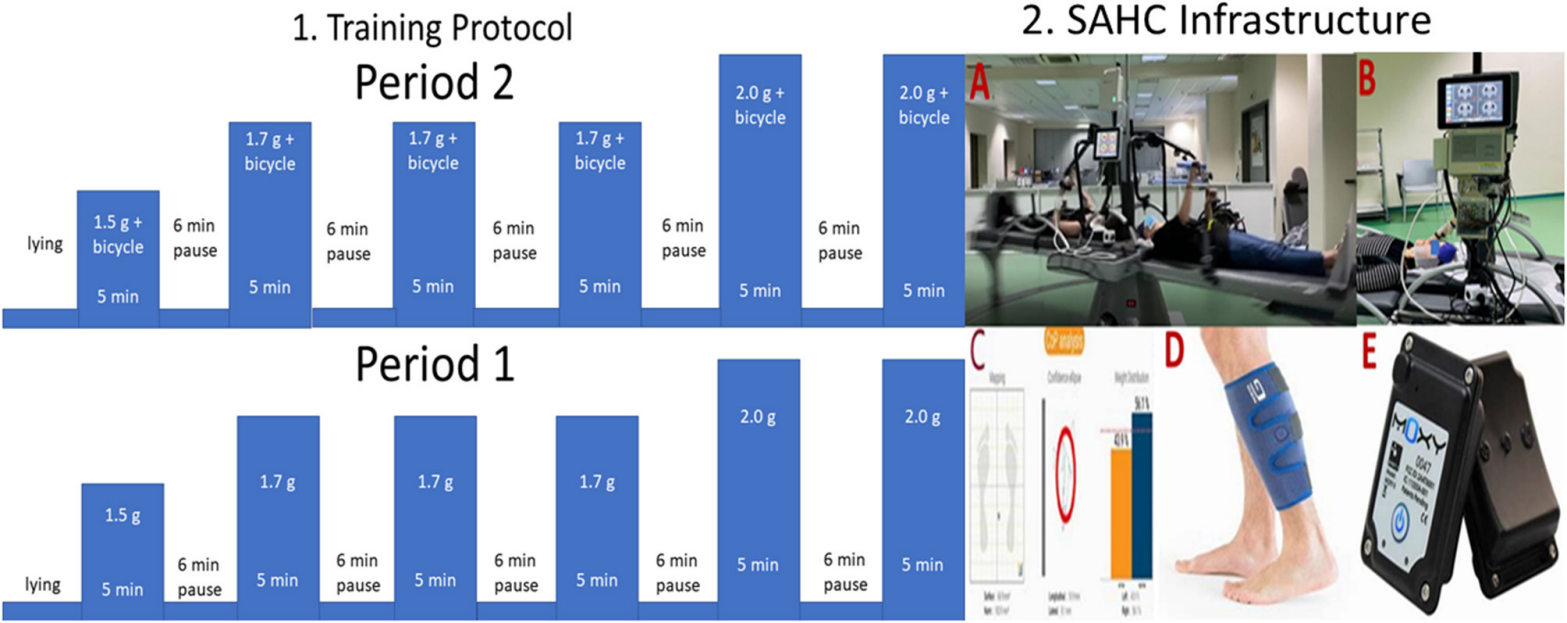
1. Visualization of the intervention protocol and 2. Visualization of the Short Arm Human Centrifuge (SAHC) infrastructure used for rehabilitation **(A)**. The infrastructure is equipped with two electroencephalographic (EEG) devices with 32 and 64 channels respectively for simultaneous neurophysiological data acquisition **(B)**. Cardiovascular biosignals were recorded every 30 s through the Clear Sight device also depicted in B. Balance outcome metrics were extracted through the Kinvent device at pre/post-intervention intervals on a standing position **(C)**. Muscle oxygenation was recorded through a near-infrared spectroscopy device **(D)**, while the device is presented in **(E)**.

The SAHC device developed by our research group (patent #1009812/13/09/2019) is located at the “Joan Vernikos” Laboratory of Aerospace and Rehabilitation Applications. As seen in Figure 3, it has a diameter of 4 m and can reach a +3.5-Gz load at the feet. It consists of a support base, while motion and rotation mechanisms such as motor, reducer, drive gear and spin gear, a shaft and two to four beds are connected to the central axis. Beds rotate extremely smoothly on wheels. The SAHC infrastructure is also equipped with several exercise systems locate on the bed extremities. Aerobic training is performed through a bicycle at one end, whose position is adjusted according to the patient's height, increasing thus the rotation radius accordingly. Resistance training is facilitated through a horizontal rowing device at the other. The entire infrastructure is connected to the electric control panel (dashboard) of the man-control room consisted of an inverter and an electronic management system (control). The SAHC infrastructure was constructed according to both national and international safety regulations, while being also applicable to patients with mobility disabilities. This issue is explained in detail in a previous study of our group (14).

#### Combination of AG Training With Physical Activity

The beneficial impact of AG training may be further enhanced through simultaneous (either aerobic or resistance) exercise. Both of them but especially the sliding system and the mechanical push/pull action of the rower, increases the gravitational load and provides an effective physical rehabilitation regime to the patients.

Aiming to investigate the clinical utility of this combined intervention we employed the followed protocol: The intervention during period 1 was based solely on centrifugation, whereas intervention during the period 2 was a combination of centrifugation with bicycle ergometer. The intervention protocol, as shown in Figure 3 (Part 1), consists of 5 min training followed by 6 min of wash-out period. The training intensity was kept between the 40%-60% of the maximum heart rate intensity, as described in (14). Almost all the muscle groups are activated during the proposed training protocol.

Through the special construction, support and rotation safety are provided. The person is laying on a horizontal position. The beds rotate up to 3.5 g at the feet, while the head is positioned close to the rotational axis and the legs outwards, simulating in this way the upright posture providing mechanical loading on all gravity sensors of the body. The use of additional (either aerobic or resistance) exercise during the rotation and especially the sliding system and the mechanical push /pull action of the rower increases the gravity load, giving effective physical rehabilitation regime to both healthy people and patients. The combination results in the feasibility of implementing different and even complicated therapeutic protocols, offering thus an unprecedented advantage through the accomplishment of a multi-system training.

### Cardiovascular Measures

Continuous cardiovascular measures were obtained through the Clear Sight (Edwards Life Sciences Corp., Irvine, CA) non-invasive monitor, attached to the participant's finger during all centrifugation sessions. Cardiac output (CO), stroke volume (SV), mean arterial pressure (MAP), heart rate (HR), systolic blood pressure (SBP), and diastolic blood pressure (DBP) were recorded.

### Muscle Oxygen Saturation

SmO_2_ of the gastrocnemius muscle of both legs was measured to provide insight into how muscles handled oxygen during the centrifugation protocol. The measurement was performed non-invasively and in real time by a portable monitoring system, insensitive to motion, the Moxy (Fortiori Design LLC, Hutchinson, MN). Moxy is a near-infrared spectroscopy device, with light-emitting diodes and photo detectors measuring the oxyhaemoglobin and oxymyoglobin contents as a percentage of the total hemoglobin and myoglobin contents in a muscle. Two Moxy devices were fixed, one to the right and one to the left gastrocnemius medialis, using opaque adhesive tape, and muscle oxygenation data were collected wirelessly by use of the Idiag Moxy software every 2 s.

### Electroencephalographic Data Acquisition

The Neurofax EEG-1200 32-channel device (Nihon Kohden, Tokyo, Japan) was used to perform (i) EEG with 19 electrodes placed according to the 10–20 International System and through bipolar electrodes (ii) electrocardiography (ECG), (iii) chin electromyography (EMG), (iv) electrooculography (EOG) for recording vertical (blinks) and horizontal eye movements. A ground electrode was placed in the prefrontal midline (Fpz) position and two reference electrodes were placed on the mastoid muscles. The data acquisition procedure included resting-state analysis while sitting (with eyes closed and open), standing and lying horizontal. Each task lasted 5 min.

#### Electroencephalographic Pre-processing

The raw EEG data were initially re-referenced to a common average reference model to facilitate further (oscillatory and connectivity) analysis. Then, a series of 2nd-order Butterworth filters were applied as follows: (a) high-pass filter with cut-off frequency at 0.5 Hz for linear trend removal, (b) low-pass filter with cut-off frequency at 50 Hz for removing high-frequency noise, (c) band-stop (notch) filter centered at 50 Hz for removing industrial noise, (d) band-stop (notch) filter centered at 100 Hz for further attenuating industrial noise harmonics that were not removed by the previous filters. Regarding the other recordings, the pre-processing steps were similar apart from some differences in filter specifications: The cut-off frequency of the low-pass filter used in ECG was set at 20 Hz. The cut-off frequency of the high-pass filter used in EMG had a cut-off frequency set at 10 Hz, while the cut-off frequency of the low-pass filter was set at 70 Hz. Finally, for EOG analysis, the cut-off frequency of the low-pass filter was set at 15 Hz.

Then, EEG data were subjected to independent component analysis by two experienced neuroscientists, who visually inspected the data to remove artifactual sources. Finally, the artifact-free data were processed in successive, non-overlapping epochs. Epoch duration was set at 16 s.

#### Electroencephalographic Analysis

EEG analysis was performed for each epoch separately. It extracted the relative energy contribution of each EEG rhythm: delta (0.5–4 Hz), theta (4–8 Hz), alpha (8–12 Hz), beta (12–20 Hz) and gamma (20–40 Hz) at both the sensor and cortical levels. It was based on the orthogonal discrete wavelet transform, as described in Frantzidis et al. (22). In brief, this analysis employs a family of biorthogonal wavelets and an iterative decomposition scheme in order to reconstruct the relative energy contribution of each rhythm with optimal time-frequency resolution.

Then, reconstruction of cortical activations was performed by using the inverse problem solution through the Brainstorm software. First, the generic head anatomy was modeled through the open M/EEG Boundary Element Method and the cortex through 15,000 dipoles of fixed orientation. Then, we estimated the cortical activity of 68 regions defined according to the Desikan-Killiany atlas (23). The cortical activity of each region was estimated as the mean time series of all dipoles included in the specific region. The computations were performed through the Matlab-based, EEGLAB (24) and the Brainstorm graphical user interfaces (25).

The cortical functional connectivity was estimated by employing the synchronization likelihood metric, a symmetric connectivity metric defined for each pair of cortical regions (26). It does not contain any information regarding the direction of the co-operation (non-directional metric) and results in a 68 × 68 metric. The main diagonal contains zero values (comparing each region with itself), while all other array cells contain a value ranging from 0 to 1. The larger the connectivity between regions, the greater the corresponding cell value is.

We focused on cortical regions showing either a linear increase or a linear decrease across the three experimental time instances. Since the intervention evaluation was performed three times (t_A_, t_B_, and t_C_), we focused on those cortical regions whose energy of rhythmic activity (delta, theta, alpha, beta and gamma) followed either a linear increase (t_A_ < t_B_ < t_C_) or a linear decrease pattern (t_A_ > t_B_ > t_C_). These cortical regions were also employed in the subsequent functional connectivity analysis, in which we also identified those following similar patterns. According to the number of functional edges, we also identified the node strength of each cortical region.

### Neurological and Physical Performance

Before and after the 4-week training programme, the following neurological and physical performance tests were carried out: The Expanded Disability Status Scale (EDSS), the Multiple Sclerosis Impact Scale 29 (MSIS-29), the Timed 25-Foot Walk Test (T25FWT) of maximal walking speed, the Symbol Digit Modalities Test (SDMT) of cognitive impairment, the 9-Hole Peg Test (9HPT) of manual dexterity and the 6-min Walk Test (6MWT) of walking endurance.

Balance was evaluated through static and dynamic posturography before and after the 1st centrifugation session. The participant stood with both legs symmetrically on two KINVENT force plates (Montpellier, France) and weight distribution difference (WDD), average position of the center of pressure (APCOP) in the medio-lateral and antero-posterior planes and average velocity of displacement (AV) were assessed with a frequency of up to 75 Hz. These outcomes were calculated with KINVENT's Balance Clinic software.

### Statistical Analysis

Statistical tests were performed using SPSS (IBM SPSS, v.25). CO, SV, MAP and HR values across the three different *g* loads (1.5, 1.7, and 2 g) of the 4-week training programme during its first two weeks (period 1) and last 2 weeks (period 2) were tested for normality using the Shapiro-Wilk test and visual inspection of the data. We then used paired *t* tests (when the normality assumption was fulfilled) and Wilcoxon signed rank test (when the normality assumption was not fulfilled) to compare changes in these parameters. Significance was set at α = 0.05. Cardiovascular data are presented as the mean ± standard deviation and are visualized through boxplots.

## Results

The primary acute and training-related intervention outcomes were (i) cardiovascular responses, (ii) SmO_2_ of the gastrocnemius medialis muscle of both legs, (iii) cortical oscillatory and functional connectivity analysis, (iv) neurological and physical performance and (v) balance. The initial validation protocol, visualized in Figure 2, provides the baseline values of the cardiovascular and muscle oxygenation parameters. Additionally, the participant was also examined at the lying position for a period of 6 min. We obtained one parameter value per minute. Their mean values are the following: CO: 7.883 ± 0.691, CI: 4.100 ± 0.341, SV: 96.667 ± 6.439, HR: 81.667 ± 5.007, SBP: 122.667 ± 15.642, DBP: 69.333 ± 6.186, MAP: 89.833 ± 9.368, Muscle Oxygenation/Right Leg: 61.383 ± 3.600, Muscle Oxygenation / Left Leg: 74.783 ± 3.347.

### Cardiovascular Responses

Analysis of the cardiovascular parameters revealed several statistically significant changes, both acutely and in response to training, as reported below.

#### Acute Exposure Responses

**Figure 4:**
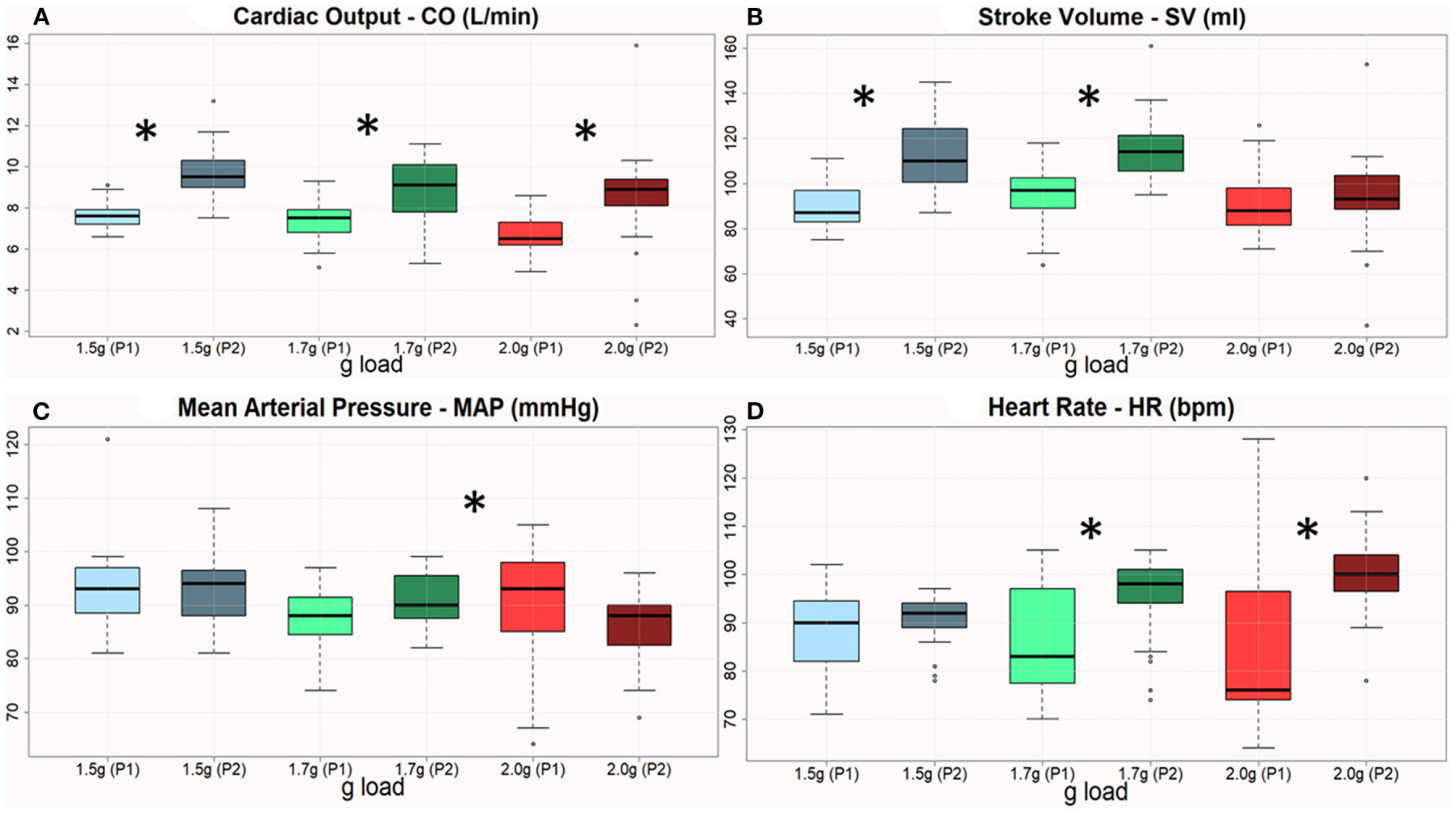
**(A–D)** Boxplots of CO, SV, MAP, and HR across 3 AG levels with respect to the 1st & 2nd period of training. The therapeutic sessions showed a significant improvement of all variables the 2nd period, except MAP in 2 g. The asterisk denotes statistically significant differences when comparing pre/post changes regarding the cardiovascular features.

Figure 4 presents CO and MAP during standing and at the *g* loads applied during the initial centrifugation test. The values closest to standing were considered optimal for prescription of the training protocol and were verified by dose-response curves, as demonstrated in our previous study (22).

#### Responses to Training

Based on the initial centrifugation validation described in the previous subsections, the clinical experts of our group developed a prescription protocol of *g* loads to be administered as follows: 1.5, 1.7, 1.7, 1.7, 2, and 2 g, each for 5 min, with 6 min of pause in between. Statistical analysis revealed significant differences in most of the cardiovascular parameters between periods 1 and 2. Most parameters improved from period 1 to period 2 in the three *g* loads employed (Figure 4).

All the four cardiovascular parameters reached statistical significance as displayed in Table 1. This Table describes the mean difference (1st−2nd month) regarding the amplitude of each cardiovascular parameter. Statistically significant changes are highlighted with blue font and reported both in terms of the p=value and the t or Z value according to the (parametric or non-parametric) test employed respectively. In case of parametric tests, we also include the degree of freedom in parentheses (here the degrees of freedom are 30).

**Table 1:** Description of the pairwise comparisons at 1st and 2nd period regarding cardiovascular features across 1.5, 1.7, and 2 g respectively.

***Cardiovascular data***	***Pairwise Comparisons***			
	1.5g	***Mean difference** **(1**st**−2**nd **month)***	***p–value***	***t(30)/Z value***
*CO*		**−1.974**	**<0.001**	**t(30)** **=** **−7.398**
*SV*		**−21.452**	**<0.001**	**t(30** **=** **−6.790**
*MAP*		−1.387	0.438	Z=- 0.775
*HR*		−2,613	0.136	Z=-1.491
	1.7g			
*CO*		**−1.565**	**<0.001**	**t(30)** **=** **−4.559**
*SV*		**−20,484**	**<0.001**	**Z** **=** **−4.567**
*MAP*		**−4.065**	**<0.001**	**t(30)** **=** **−4.013**
*HR*		**−9.258**	**0.002**	**Z** **=** **−3.106**
	2g			
*CO*		**−1.826**	**<0.001**	**Z** **=** **−3.646**
*SV*		−3.452	0.220	Z=-1.225
*MAP*		+3.678	0.073	Z=-1.796
*HR*		**−16.097**	**<0.001**	**Z** **=** **−4.033**

*Bold values indicate statistically significant differences among outcome measures at pre/post level*.

### Oxygen Saturation of the Gastrocnemius Medialis Muscles (SmO_2_)

**Figure 5:**
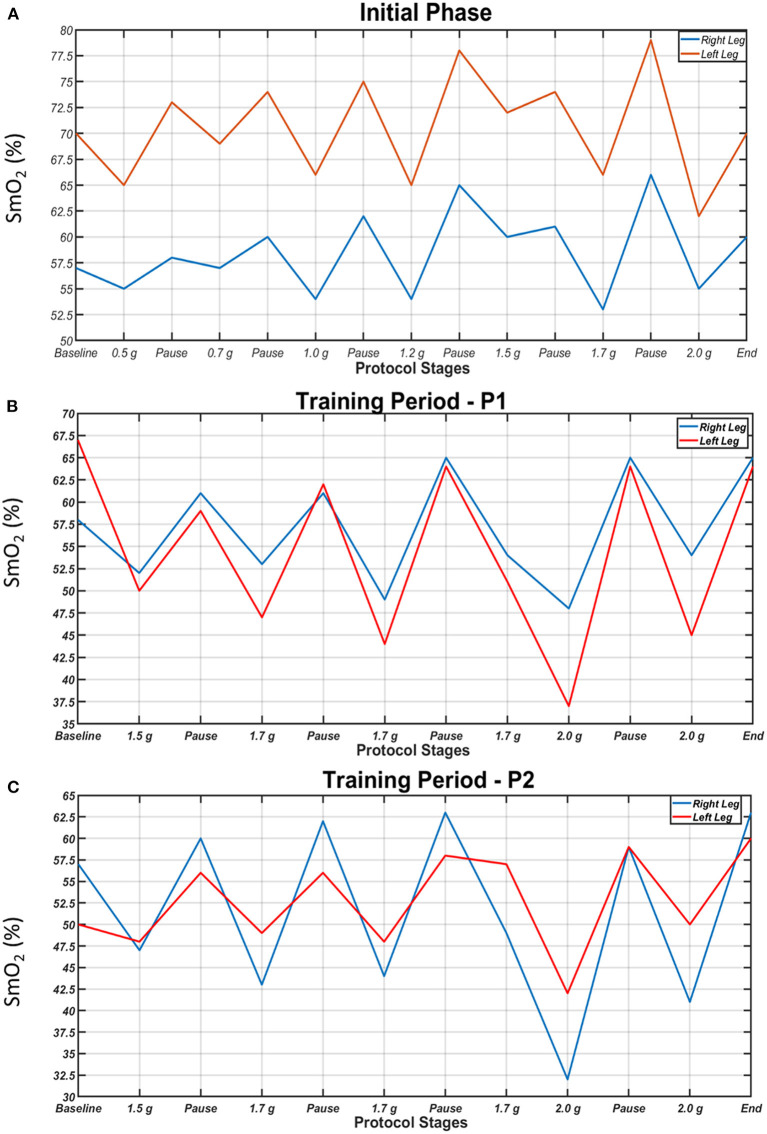
Oxygen saturation of the gastrocnemius medialis muscles (SmO_2_) of the right (blue) and left leg (red) during each step of the initial centrifugation test **(A)**, a session in the 1st period **(B)** and a session in the 2nd period of the study **(C)**. SmO_2_ dropped during centrifugation and increased during pauses. Higher g values generally resulted in lower SmO_2_.

SmO_2_ in the two legs during the initial centrifugation test, a session in the 1st period and a session in the 2nd period are presented in Figure 5. In all cases and in both legs, SmO_2_ decreased during centrifugation and increased during pauses. In general, the higher the *g* value, the lower the SmO_2_ was. Additionally, the difference in SmO_2_ between legs tended to decrease with the progression of centrifugation sessions due to a decrease in oxygenation of the suffering (left) leg, indicative of increased oxygen utilization with training.

### Cortical Oscillatory and Functional Connectivity Analysis

**Figure 6:**
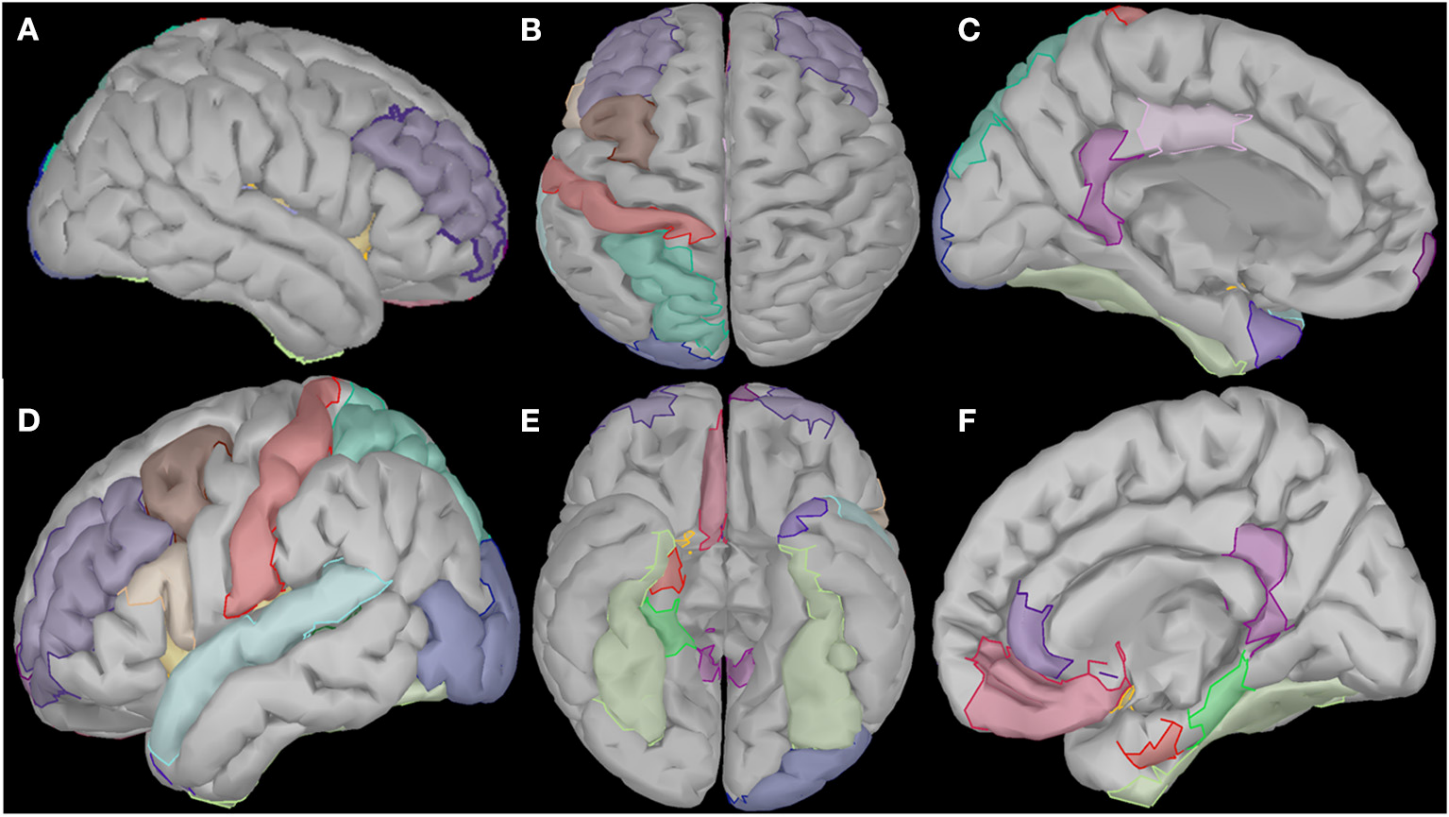
Visualization of the cortical regions that changed linearly (either increased or decreased) from the beginning to the end of the intervention period for all frequency bands (delta, theta, alpha, beta and gamma). These regions diffuse in several cortical and subcortical areas such as right prefrontal and temporal areas **(A)**, central and posterior areas **(B)**, temporal and subcortical areas of the left hemisphere **(C)**, prefrontal, temporal and occipital areas of the right hemisphere **(D)**, and several subcortical areas of both hemispheres **(E,F)**.

Cortical oscillatory analysis was employed during a standing task lasting 5 min. The oscillatory features were extracted as the mean values of the relative energy contribution of the EEG rhythms across all 16-s epochs. We identified 23 cortical regions that showed linear increases (for delta, theta and alpha) and simultaneously linear decreases (beta and gamma) from the beginning to the end of the intervention period (Figure 6). We included only those showing the aforementioned patterns for each frequency band. Table 2 displays the regions' names and centroid coordinates in the Montreal Neurological Institute space.

**Table 2:** Name and description of the cortical regions that changed linearly across the intervention and frequency bands.

**Name**	**Lobe**	**Hemisphere**	**MNI co-ordinates**		
			**X**	**Y**	**Z**
Banks of superior temporal sulcus	Temporal	Left	−64.8	−48.5	3.2
Caudal middle frontal	Frontal	Left	−36.4	18.5	57.6
Entorhinal	Temporal	Right	21.2	−5.1	−35.4
Frontal Pole	Frontal	Right	7.1	64.6	−9.3
Fusiform	Temporal	Left	−27.8	−40.4	−23.0
		Right	32.0	−40.6	−24.2
Insula	Temporal	Left	−28.3	5.0	−19.2
		Right	21.9	2.2	−12.7
Isthmus cingulate	Limbic	Left	−4.8	−45.2	14.1
		Right	1.6	−44.4	15.8
Lateral occipital	Occipital	Left	−25.0	−103.1	−2.1
Medial orbitofrontal	Frontal	Right	−3.3	45.7	−25.5
Parahippocampal	Temporal	Right	18.0	−33.4	−17.2
Pars opercularis	Frontal	Left	−62.5	11.3	19.8
Postcentral	Central	Left	−52.3	−19.6	56.8
Posterior cingulate	Limbic	Left	−4.1	−36.2	50.5
Rostral anterior cingulate	Limbic	Right	3.2	13.7	−9.6
Rostral middle frontal	Frontal	Left	−38.6	48.5	18.3
		Right	33.6	51.1	8.6
Superior parietal	Parietal	Left	−13.5	−73.0	57.4
Superior temporal	Temporal	Left	−63.8	−13.0	1.2
Temporal pole	Temporal	Left	−21.3	17.4	−38.8
Transverse temporal	Temporal	Right	55.7	−9.2	8.0

*The regions are reported according to their name, their anatomical lobe and hemisphere and their Montreal Neurological Institute (MNI) co-ordinates*.

Cortical regions were divided by their anatomical position in three cortical groups (frontal, temporal and posterior/centro-parieto-occipital) and in one limbic group, with the widest one being the temporal group (consisting of 10 regions), followed by the frontal one with six regions (one prefrontal). The limbic group contains four regions and the posterior one, only three (left hemisphere). Details about their anatomical positions may be found in Table 2.

In frontal regions, we observed changes in left caudal middle frontal and left rostral middle frontal gyri, as well as the right superior frontal gyrus and the left medial frontal gyrus. In limbic regions, changes were observed in the right parahippocampal gyrus and the right entorhinal cortex. In the insular cortex, we observed increased activation of the left and right insula. The temporal lobe and, specifically, the left superior temporal gyrus, the bilateral fusiform gyri and the left banks of superior temporal sulcus revealed spectral cortical differences. In occipital regions, we saw changes in the posterior region along the lateral occipital cortex. Finally, in the parietal regions, changes were observed in the postcentral gyrus and the superior parietal gyrus.

**Figure 7:**
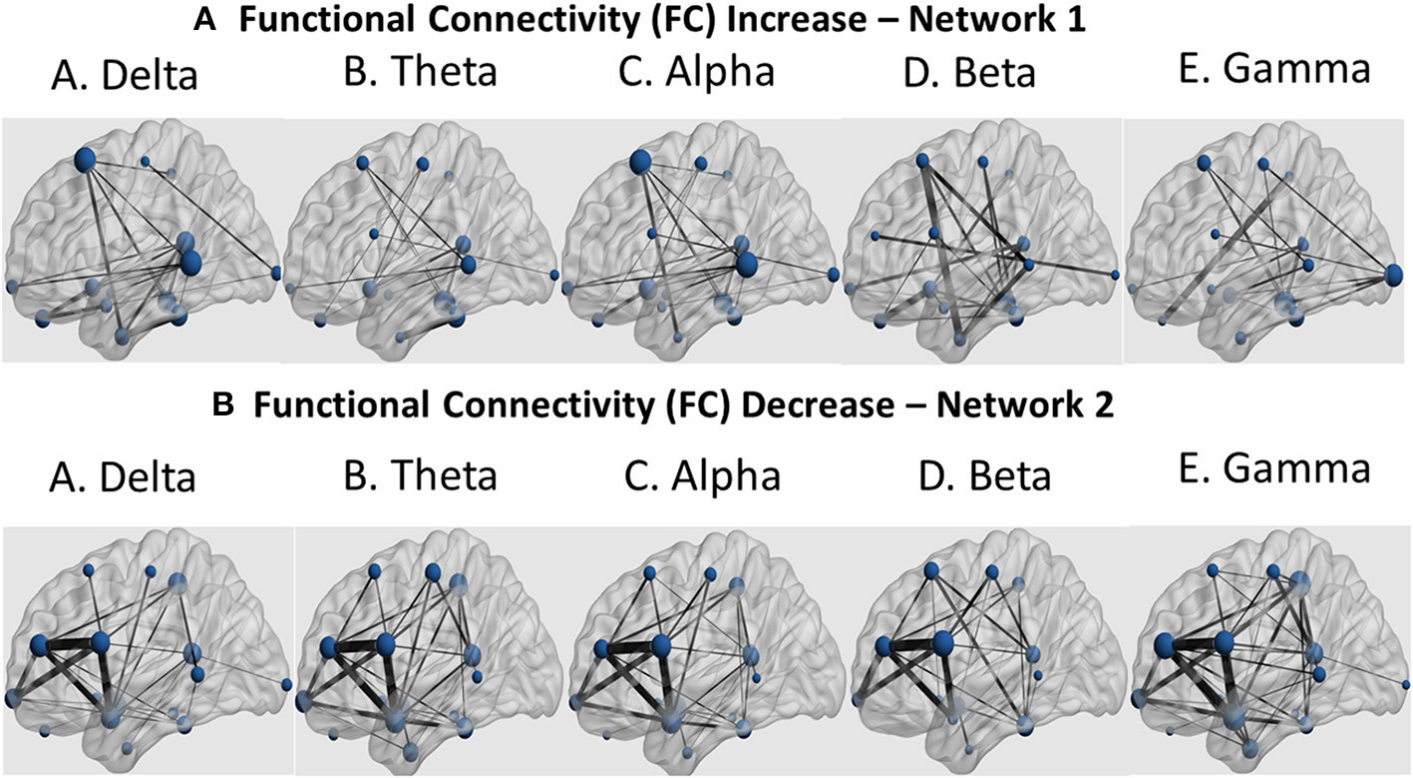
Sagittal view of the two subnetworks showing either a functional connectivity linear increase **(A)** or decrease pattern **(B)** across the three experimental stages (baseline, 1st and 2nd training periods) for the five cortical rhythms (delta, theta, alpha, beta and gamma) among the cortical regions identified by the cortical oscillatory analysis. The node size is proportional to its degree, whereas the edge width is proportional to the connectivity magnitude.

**Figure 8:**
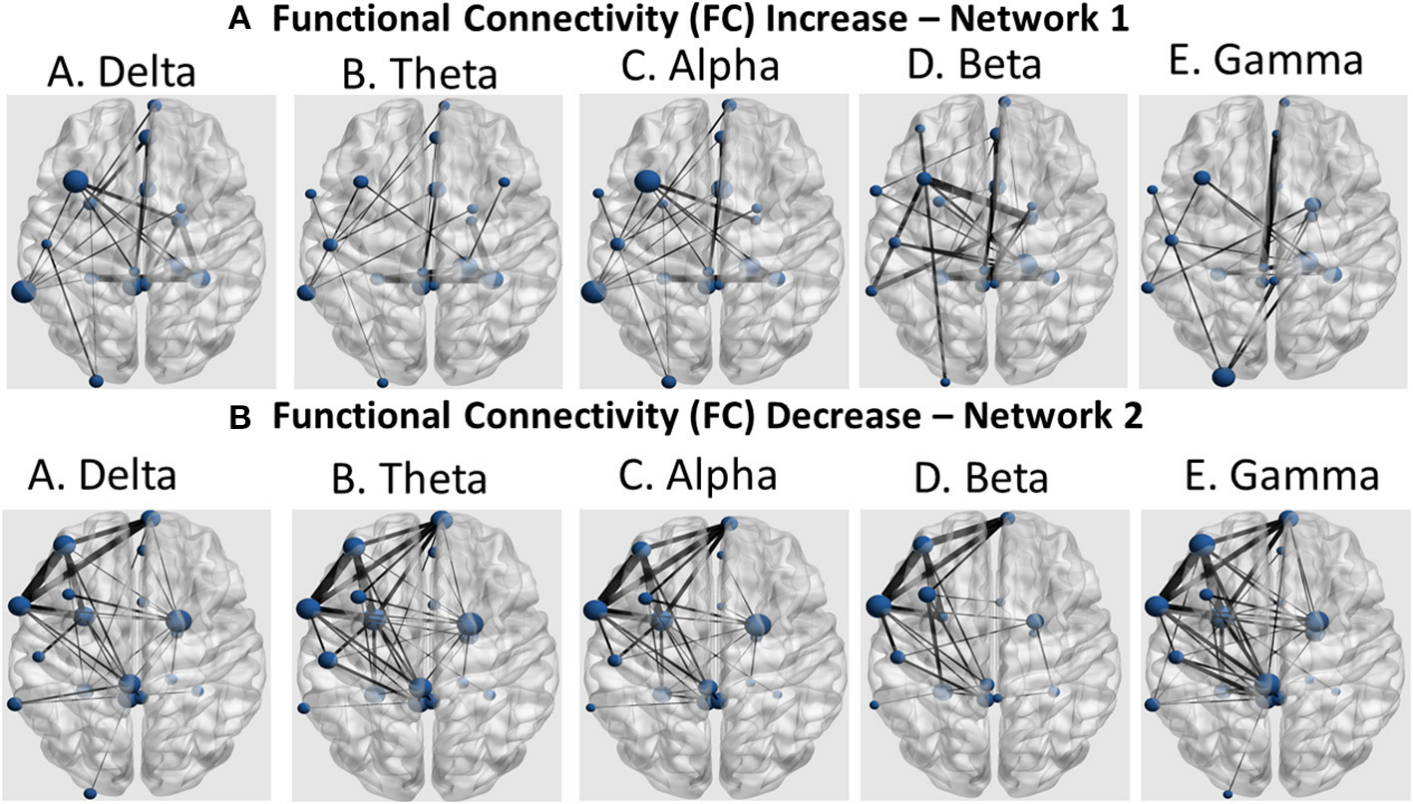
Coronal view of the two subnetworks showing either a functional connectivity linear increase **(A)** or decrease pattern **(B)** across the three experimental stages (baseline, 1st and 2nd training periods) for the five cortical rhythms (delta, theta, alpha, beta, and gamma) among the cortical regions identified by the cortical oscillatory analysis. The node size is proportional to its degree, whereas the edge width is proportional to the connectivity magnitude.

Further identification among the aforementioned cortical regions revealed two cortical subnetworks. The first one, described in Figures 7A, 8A, exhibited a linear functional connectivity increase during the various experimental stages. Simultaneously, there was another subnetwork (Figures 7B, 8B) demonstrating functional connectivity decrease when comparing the baseline condition with the 1st and 2nd training periods. For both networks, the size of the node is indicative of its node degree, while the edge width represents the magnitude of the connectivity change in absolute terms.

#### 3.4. Neurological and Physical Performance

The 4-week programme improved performance in all neurological and mobility tests. Specifically, the EDSS score dropped from 6 to 5 and the MSIS-29 score from 66 to 59. The T25FW test was completed in 14.15 s before and in 11.14 s after the programme. The correct number of substitutions in 90 s, in the SDMT was 30 before and 49 after the training programme. The 9HPT score, being 33 s for the right hand and 26 s for the left hand before, dropped to 26 s and 23 s, respectively, after training. The distance covered in the 6MWT was 152 m before and 195 m after the programme.

Adhesion to and tolerability of the intervention were assessed by the 10-point Motion Sickness Symptom Scale and the 5-point exit survey administered at the end of each session Tables 3, 4, respectively). Intermittent centrifugation was well tolerated by the patient in terms of cardiovascular loading and motion sickness during the entire study. No adverse effects or unanticipated events were observed.

**Table 3:** Motion sickness symptom scale.

**Rating**	**Motion sickness symptoms**
0 √	No symptoms
1	Any symptom no matter how slight
2	Minimal warmth, fatigue
5	Stomach awareness
7	Moderate nausea
9	Incipient vomiting
10	Vomiting

**Table 4:** Exit survey.

**Question 1**	How comfortable exercise was on the centrifuge (1 = very uncomfortable to 5 = very comfortable)	5
**Question 2**	How difficult the resistance exercise was on the centrifuge (1 = very easy to 5 = very difficult)	2
**Question 3**	How sore do you feel in your muscles and body? (1 = no effect to 5 = severe)	2
**Question 4**	How severe motion sickness symptoms did you feel on the centrifuge? (1 = no symptoms to 5 = severe symptoms)	1
**Question 5**	How severe did you feel the effect of Coriolis force on your knees during centrifugation? (1 = no effect to 5 = severe effect)	1

Regarding balance, WDD, APCOP in the medio-lateral and antero-posterior planes, as well as AV decreased after, compared to before, the 1st centrifugation session (from 3.9 to 0.4 %, from 98.8 to 82.0 mm and from 10.1 to 8.4 mm/s, respectively).

Although the aforementioned outcome measures offer valuable physiological information, their absolute values representing the difference at pre-post intervention level should be interpreted with caution. Therefore, Table 5 includes the percentage ratio of improvement.

**Table 5:** Improvement percentage (%) of the outcome measures after treatment.

**Outcome type**	**Feature**	**Improvement percentage (%)**
Cardiovascular	Cardiac output (2.0g)	21.40
	Stroke volume (1.5g)	19.35
	Mean arterial pressure (1.7g)	4.46
	Heart rate	14.13
Muscle oxygenation	Leg difference attenuation during baseline	50.00
Neurological assessment	Expanded disability status scale (EDSS)	33.00
	Multiple sclerosis impact scale 29 (MSIS-29)	10.61
	Symbol digit modalities test (SDMT) of cognitive impairment	38.78
	Timed 25-foot walk test (T25FWT) of maximal walking speed	21.27
	9-Hole peg test–left hand (9HPT)	21.21
	9-Hole peg test–right hand (9HPT)	11.54
	6-minute walk test (6MWT) of walking endurance	22.05
Cortical functional connectivity	Temporal regions	44.78
	Frontal regions	41.05
	Limbic regions	45.86
	Posterior regions	36.71

## Discussion

Our main finding is a multi-system effect of the gravitational load on an MS patient, validating the hypothesis that artificial gravity challenges (27), may be considered as a new rehabilitation tool to counter the detrimental consequences of MS deconditioning, since MS impairments resulting from deconditioning, are often reversible with exercise (27). As seen in Table 5, the exercise-mimicking effect of centrifugation on the patient's body was documented by the (i) cardiovascular increases (21.40% in CO during 2.0 g, 19.35% in SV during 1.5 g, 4.46% in MAP during 1.7 g and 16.13% in HR during 2.0 g), (ii) muscle oxygenation (50% attenuation of leg differences during baseline), (iii) neurological and physical performance, (iv) balance and (v) brain cortical oscillation and functional connectivity responses measured. More specifically, the improvement percentage at the end of the intervention intervals were 33% for the EDSS, 10.61% for the MSIS-29, 38.78% for the SDMT, 21.21% for the left and 11.54% for the right hand regarding the 9HPT, 22.05% for the 6MWT and 21.27% for the T25PW. There was also an average 44.78% increase in the temporal cortical regions, 41.05% increase in the frontal cortical regions, 45.86% increase in limbic and 36.71% increase in cortical posterior (parieto-occipital) regions. The aforementioned results were observed at the end of Period 2. Although there were improvements even at the end of Period 1, these were magnified at the end of Period 2. This was mainly the case for the cortical functional connectivity alterations which followed a linear pattern of improvement among the three experimental phases (Baseline, Period 1, Period 2). So, this study provides first evidence that the combination of artificial gravity with aerobic training may be more robust than simple aerobic or AG training.

The core research question answered by the present study, was the definition of the most appropriate centrifugation load, for optimal intervention outcomes. By employing a complete set of optimal cardiovascular indices (CO, MAP, HRV, HR) followed during training, combined with the information received from simultaneous recording of the patient's SmO_2_ indicating the lower drop level where the maximum exercise is achieved, before the start of muscle fatigue, may lead to the maximum benefit of the combination of centrifugation with exercise; in our case study, the most appropriate g load to start training was shown to be 1.5 g.

### Cardiovascular Responses

The improvement in CO and HR over time (Figure 2) showed the effectiveness of the training programme in improving the patient's physical condition. This is in accordance with the results of the study of Rampello et al. (28), who demonstrated that cardiorespiratory training is better than neurorehabilitation in improving functional and moving capacity in MS patients with EDSS <7. The proposed intervention facilitates cardiovascular adaptation, in accordance with previous knowledge (29), suggesting that the observed HR response may be attributed to deconditioning rather than autonomic dysfunction *per se*. The increasing CO over time indicated beneficial adaptation to acceleration. Following the intervention, SV also increased (Table 1), an adaptation also documented in response to aerobic exercise programs (30). Our findings indicate a cardiovascular challenge during centrifugation, introducing a new stressor on the heart. This, initially causes a BP drop due to the displacement of the body fluids toward the lower extremities, with immediate reflex calf muscle constriction, functioning as a pump to send blood back to the brain (Figure 2) (31). Initially, muscle oxygen saturation presented a difference between the non-dominant (right) and the dominant leg (Figure 5). We noticed higher power on the left leg during centrifugation, maybe due to a higher consumption. Then, an increase in HR, MAP (Table 1), SBP and DBP followed (SMF9), attributed to the body response to the pressure reduction by increasing sympathetic stimulation, to aid venous return and return blood pressure to nominal levels at the baroreceptors, findings similar to those of healthy populations (32).

### Muscle Oxygenation

One of the main effects of the centrifugation training programme on the MS case was the near elimination of the initial difference in gastrocnemius medialis muscle oxygenation between legs through a decrease in oxygenation of the suffering leg, suggestive of increased oxygen use during centrifugation. During the first steps of the initial centrifugation test, when *g* load was low (0.5–1 g), changes in SmO_2_ were smaller, compared to the main centrifugation sessions, in which higher *g* loads were applied (Figure 5). The observed consistent decrease in SmO_2_ with the application of *g* load, compared to the pause, is comparable to decreased muscle oxygenation during hard exercise (33, 34). This shows that the amount of oxygen supplied to the muscles was less than that being utilized in them, possibly due to increased consumption as a result of muscle activity to counteract the gravitational load. Our findings that the higher the *g* load, the lower the SmO_2_ and that centrifugation training increased oxygen use in the suffering limb introduces centrifugation as a robust exercise rehabilitation device in MS.

### Neurological and Physical Performance

The improvement in all neurological and mobility tests with the training programme applied shows the effectiveness of centrifugation in ameliorating functional capacity, which is of paramount importance in everyday activities of MS patients. Additionally, the decrease in WDD, APCOP and AV after the application of the therapeutic protocol suggests a positive effect of SAHC on static balance in MS.

### Oscillatory Activity and Functional Connectivity

Sensorimotor brain network areas are generally decreased in people with MS (35). We observed changes in different regions of the brain after the intervention, intercorrelated with improvements in cardiorespiratory, muscular and visuospatial function, as well as cognitive benefits.

In frontal regions, we observed changes in left caudal middle frontal and left rostral middle frontal gyri (Figures 7, 8), as usually seen in MS patients. The same lobe with the right superior frontal gyrus and the left medial frontal gyrus revealed increased functional connectivity with thalamus, resembling exercise training-induced neuroplasticity, which intercorrelated with improvements in cardiorespiratory fitness of the same patients (36). Frontal pole activation was also described by Zanchi et al. (37), highlighting the frontal pole as a region of better motor outcomes. Activation of the left pars opercularis and the right medial orbitofrontal cortex, both being parts of the inferior frontal gyri, was observed with resistance training-related improvements in grip strength of the MS patient, with similar results described by Akbar et al. (38), along with increased resting state functional connectivity in bilateral inferior frontal regions and the left middle frontal region. Activation and increased connectivity of the same area, and specifically the bilateral postcentral gyri of MS patients, were attributed by Tavazzi et al. (39), to the combined aerobic and resistance intervention, which resulted in enhanced resting state functional connectivity in sensorimotor brain network areas, as well as improved balance, walking endurance and gait.

In limbic regions, changes observed in the right parahippocampal gyrus were similar to those reported previously, where structural changes and volume increases occurred after dance training (40). The changes observed in the right entorhinal cortex were an important finding of our study, as this region is regarded the mediator of the hippocampus and several cortical regions involved in memory processing, especially in the case of visuospatial information (41). Animal studies also suggested that long-term running induces neuroplasticity in both hippocampus and the entorhinal cortex (42).

In the insular cortex, we observed increased activation of the left and right insula, a region previously associated with cortical control of cardiovascular alterations due to physical training and autonomic arousal (43). It seems that exercise-induced insular activation is proportional to the extent of exercise and stress, quantifying thus the amount of motor neuroplasticity (44).

The temporal lobe and, specifically, the left superior temporal gyrus revealed spectral cortical differences after the intervention. This is the region known for its plasticity in response to aerobic exercise (45). Other regions of the same lobe that exhibited spectral power changes were the bilateral fusiform gyri, areas malleable to motor function rehabilitation (46). Our findings also extended to the left banks of superior temporal sulcus, which is a relatively unexplored region known to be involved in biological motion perception (47). Thus, the findings in those two temporal regions may be an indication that artificial-gravity training may enhance motion accuracy through advanced motion perception and visual formation of the motor tasks. The temporal pole was also found by other researchers to be plastic to motor training and sport expertise (47). The last temporal region in which regional volumes of cortical and subcortical gray matter changed in response to the intervention applied in the present study was the transverse temporal gyrus, which has been associated with self-reported physical activity and brain age (48).

In occipital regions, we have revealed changes in the posterior part along the lateral occipital cortex, in accordance with studies where occipital activation was observed in inactive individuals after exercise interventions (49). Our finding may be also in line with a study demonstrating decreased gray matter within the lateral occipital cortex, which was considered as a biomarker for physical frailty and cognitive impairment in patients with Parkinson's disease (50).

We also observed alterations in the postcentral gyrus and the superior parietal gyrus after the centrifugation intervention, which is reminiscent of exercise-induced cognitive performance improvement. Specifically, a previous study reported functional activation of the left angular gyrus in patients over 35 years old. Shorter-term exercise interventions (under 12 weeks) primarily induced changes in right occipital, parietal and limbic regions, while longer-term interventions (12 weeks or more) activated the parietal lobe encompassing the angular and supramarginal gyrus (49). Similarly, gray matter volume within the postcentral and superior parietal gyri are correlated with the level of self-reported physical activity (51). So, these findings may provide markers of physical activity level.

The identified cortical and limbic regions (left and right isthmus cingulate, left posterior cingulate cortex and right rostral anterior cingulate cortex) have been found to be vulnerable to neurodegenerative effects associated with pathologic aging (52) but also more activated in physically active MS patients compared to inactive patients. Regarding the isthmus cingulate, studies consistently report a reduction in fractional anisotropy of the tracts including the genus of the corpus callosum in MS patients relative to healthy controls (53). The posterior cingulate gyrus has also been shown to be activated by short-term exercise interventions (49).

We observed widespread alterations in cortical rhythmic activity and functional connectivity during standing. These are summarized in a linear increase of slow cortical rhythms (delta and theta) and a linear decrease of the fast rhythms (alpha, beta and gamma). Then, we used these regions in a functional connectivity analysis to demonstrate a similar pattern. These seed regions, observed mainly in frontal, temporal, posterior and limbic areas, modified their rhythmic activity during the study. According to Bamidis et al. (6), novel environmental stimulation may serve as the stimulus for neuroplasticity induction. Previous neuroscientific evidence demonstrated that spectral functional connectivity markers may serve as a reliable guide for neurofeedback interventions to alleviate fatigue (54). Our analysis also employs cortical connectomics to identify neuroplastic effects due to the proposed intervention.

### Limitations and Further Study

We should also clarify that when the MS patient enrolled in the study was already under physiotherapy and exercise, which was not enough to enhance his mobility. When the intervention initiated, the improvement became obvious and especially when centrifugation was combined with both endurance and resistance exercise. Although the longitudinal design of our study clearly demonstrates the validity of the proposed SAHC infrastructure as a methodological framework for novel rehabilitation approaches, the beneficial impact of centrifugation on the MS pathology may be further validated through forthcoming randomized controlled trials. Within the context of the H2020-funded project VITALISE (55), we aim to promote co-operation within several laboratories focusing on rehabilitation and to establish common tools applied to clinical settings. First, the study design should be further validated by a randomized controlled trial which would compare MS patients randomly assigned either to the proposed intervention or to a passive control group. This would provide the scientific community with the required statistical evidence needed to support the feasibility of the proposed intervention. The proposed intervention should also be compared with other existing state-of-the-art intervention approaches, in order to investigate whether it induces an additional benefit for the patients. Follow-up periods should also be included to provide evidence for the sustainability of the improvement. Optimal intervention dosages in terms of intensity and duration should be also investigated for each patient through a precision medicine perspective. We wish also to further improve the infrastructure capacity by integrating centrifugation with decision making systems that would receive physiological features from sensorial data in order to offer the clinicians the feasibility to dynamically adjust the intervention parameters. Sensors would also facilitate the quantification of the exercise intensity by estimating the exertion forces and the patients' cardiovascular effort through time-frequency heart rate variability analysis. Therefore, we plan to integrate sensors for estimating systemic vascular resistance, heart-rate variability and exercise intensity. The present neurophysiological analysis was focused only on the standing condition measured at three different experimental instances toward the evaluation of the intervention efficacy regarding the two experimental periods. Moreover, neuroplasticity effects may be further investigated through blood biomarkers such as brain derived neurotrophic factor and other growth factors. Neuroimaging and high-density electroencephalography (EEG) or MEG/fMRI examinations would further strengthen our findings. So, our forthcoming research activities would employ a high-density EEG data acquisition at pre/post-intervention phases and periodical testing with the current device every 10 sessions with simultaneous centrifugation. This new evaluation approach is hypothesized to provide further insight into how artificial gravity and physical training is beneficial in clinical rehabilitation protocols.

## Conclusion

This clinical case study provides the first evidence for the rehabilitative capacity of artificial-gravity training. The idea stems from the proven efficacy of artificial-gravity training in preserving multi-system capacity of astronauts, which is proportional to the deconditioning effect of chronic immobilization. The study results provide novel insight into the beneficial impact of centrifugation on the pathology of MS. The improvement of cardiovascular, neurological and physical performance variables, in conjunction with the appearance of new brain cortical connections and the decrease in beta and gamma cortical oscillatory activity, supports the prescription of the therapeutic protocol. Additionally, the decrease in disability decrease was supported accompanied by the appearance of new brain cortical connections, as well as the decrease of beta and gamma cortical oscillatory activity and EDSS decrease. Functional connectivity alterations on two distinct networks involving both cortical and limbic regions, were associated with the exercise-induced improvements during three experimental stages (baseline, 1st and 2nd training periods). On that account, we could assume that this cognitive-motor coupling reflects a neuroplasticity effect. To sum up, the proposed intervention transfers knowledge from the space sector to the clinical rehabilitation routine.

## Data Availability Statement

The original contributions presented in the study are included in the article/supplementary material, further inquiries can be directed to the corresponding author.

## Ethics Statement

The studies involving human participants were reviewed and approved by Bioethics Committee of the School of Medicine of the Aristotle University of Thessaloniki (179/19.03.2020). The patients/participants provided their written informed consent to participate in this study.

## Author Contributions

CK-P, JV, and CF conceived and designed the research. CK-P, CN, AP, AK, IM, and CF conducted the experiments. CF and SG analyzed the data. AP, NK, and VM performed the balance analysis. SG performed the statistical analysis. CF and IM developed the neurophysiological pre-processing & analysis pipeline. CF, IM, and CN performed the neurophysiological analysis. CF, CK-P, and AK designed the figures. CB, ED, CK-P, EB, and AK performed the medical and neurological examinations. CK-P, CF, AK, and SG wrote the manuscript. AP, AK, VM, PB, EB, CK-P, JV, and CF revised the manuscript. PB and JV supervised the study. All authors contributed to the article and approved the submitted version.

## Funding

This study was funded by the European Union Horizon 2020 Research and Innovation Programmmes VITALISE (No. 101007990 - https://vitalise-project.eu/) and URBANOME (No. 945391 - https://www.urbanome.eu/).

## Conflict of Interest

JV is the director and founder of the ThirdAge company. The remaining authors declare that the research was conducted in the absence of any commercial or financial relationships that could be construed as a potential conflict of interest.

## Publisher's Note

All claims expressed in this article are solely those of the authors and do not necessarily represent those of their affiliated organizations, or those of the publisher, the editors and the reviewers. Any product that may be evaluated in this article, or claim that may be made by its manufacturer, is not guaranteed or endorsed by the publisher.

## References

[1] Friese MA Schattling B Fugger L. Mechanisms of neurodegeneration and axonal dysfunction in multiple sclerosis. Nat Rev Neurol. (2014) 10:4–225. 10.1038/nrneurol.2014.37 24638138

[2] Khan O Leist TP Vollmer TL Zamvil SS. Investigating multiple sclerosis: targeting therapeutic options. Int J MS Care. (2008) 10:5–20. 10.7224/1537-2073-10.S2.133757581

[3] Mahalakshmi B Maurya N Lee SD Bharath Kumar V. Possible neuroprotective mechanisms of physical exercise in neurodegeneration. Int J Mol Sci. (2020) 21:16–5895. 10.3390/ijms21165895 32824367 PMC7460620

[4] Negaresh R Motl RW Zimmer P Mokhtarzade M Baker JS. Effects of exercise training on multiple sclerosis biomarkers of central nervous system and disease status: a systematic review of intervention studies. Eur J Neurol. (2019) 26:711–21. 10.1111/ene.13929 30734989

[5] Kalb R Brown TR Coote S Costello K Dalgas U Garmon E . Exercise and lifestyle physical activity recommendations for people with multiple sclerosis throughout the disease course. Mult Scler. (2020) 26:1459–69. 10.1177/1352458520915629 32323606 PMC7575303

[6] Bamidis PD. Vivas A?, Styliadis C, Frantzidis C, Klados M, Schlee W, Siountas A, Papageorgiou SG. A review of physical and cognitive interventions in aging. Neurosci Biobehav Rev. (2014) 44:206–20. 10.1016/j.neubiorev.2014.03.019 24705268

[7] Edgerton VR Roy RR. Neuromuscular adaptation to actual and simulated weightlessness. Adv Space Biol Med. (1994) 4:33–67. 10.1016/S1569-2574(08)60134-3 7757253

[8] Galloway MT Lalley AL Shearn JT. The role of mechanical loading in tendon development, maintenance, injury, and repair. J Bone Joint Surg Am. (2013) 95:1620–28. 10.2106/JBJS.L.01004 24005204 PMC3748997

[9] Gupta R Truong L Bear D Chafik D Modafferi E Hung CT. Shear stress alters the expression of myelin-associated glycoprotein (MAG) and myelin basic protein (MBP) in Schwann cells. J Orthop Res. (2005) 23:1232–9. 10.1016/j.orthres.2004.12.010 16140204

[10] Kourtidou-Papadeli C Papadelis C Vernikos J Bamidis PD Hitoglou-Antoniadou M Perantoni E . The therapeutic benefits of gravity in space and on earth. Hippokratia. (2008) 12:28. 19050751 PMC2577396

[11] Clément G Pavy-Le Traon A. Centrifugation as a countermeasure during actual and simulated microgravity: a review. Eur J Appl Physiol. (2004) 92:235–48. 10.1007/s00421-004-1118-1 15156322

[12] Voss MW Nagamatsu LS Liu-Ambrose T Kramer AF. Exercise, brain, and cognition across the life span. J Appl Physiol. (2011) 111:1505–13. 10.1152/japplphysiol.00210.2011 21527670 PMC3220305

[13] Lublin FD Reingold SC Cohen JA Cutter GR Sørensen PS Thompson AJ . Defining the clinical course of multiple sclerosis: the 2013 revisions. Neurology. (2014) 83:278–86 10.1212/WNL.0000000000000560 24871874 PMC4117366

[14] Kidgell DJ Pearce AJ. What has transcranial magnetic stimulation taught us about neural adaptations to strength training? A brief review. J Strength Cond Res. (2011) 25:3208–17. 10.1519/JSC.0b013e318212de69 21993027

[15] Kourtidou-Papadeli C Frantzidis CA Gylou S Plomariti CE Nday CM Karnaras D . Gravity threshold and dose response relationships: health benefits using a short arm human centrifuge (SAHC). Front Physiol. (2021) 12:612. 10.3389/fphys.2021.644661 34045973 PMC8144521

[16] Yang CB Zhang S Zhang Y Wang B Yao YJ Wang YC . Combined short-arm centrifuge and aerobic exercise training improves cardiovascular function and physical working capacity in humans. Med Sci Monit. (2010) 16:575–83. 21119574

[17] Vernikos J Ludwig DA Ertl AC Wade CE Keil L O'Hara D. Effect of standing or walking on physiological changes induced by head down bed rest. Implications for spaceflight. Aviat Space Environ Med. (1996) 67:1069–79. 8908346

[18] Janmey PA McCulloch CA. Cell mechanics: integrating cell responses to mechanical stimuli. Annu Rev Biomed Eng. (2007) 9:1–34. 10.1146/annurev.bioeng.9.060906.151927 17461730

[19] Genchi GG Rocca A Marino A Grillone A Mattoli V Ciofani G. Hypergravity as a tool for cell stimulation: implications in biomedicine. Front Astron Space Sci. (2016) 3:26. 10.3389/fspas.2016.00026

[20] Korolkov VI Kozlovskaya IB Kotovskaya AR Krotov VP Vil-Viliams IF Lobachik VI. Efficacy of periodic centrifugation of primates during 4-week head-down tilt. Acta Astronaut. (2001) 49:237–42. 10.1016/S0094-5765(01)00102-3 11669113

[21] Pead MJ Skerry TM Lanyon LE. Direct transformation from quiescence to bone formation in the adult periosteum following a single brief period of bone loading. J Bone Miner Res. (1988)3:647–56. 10.1002/jbmr.5650030610 3251399

[22] Frantzidis CA Vivas AB Tsolaki A Klados MA Tsolaki M Bamidis PD. Functional disorganization of small-world brain networks in mild Alzheimer's Disease and amnestic Mild Cognitive Impairment: an EEG study using Relative Wavelet Entropy (RWE). Front Aging Neurosci. (2014) 6:224. 10.3389/fnagi.2014.00224 25206333 PMC4144118

[23] Desikan RS Ségonne F Fischl B Quinn BT Dickerson BC Blacker D Buckner R Dale AM Maguire RP Hyman BT Albert MS Killiany RJ. An automated labeling system for subdividing the human cerebral cortex on MRI scans into gyral based regions of interest. Neuroimage. (2006) 31:968–80. 10.1016/j.neuroimage.2006.01.021 16530430

[24] Delorme A Makeig S. EEGLAB: an open source toolbox for analysis of single-trial EEG dynamics including independent component analysis. J Neurosci Methods. (2004) 134:9–21. 10.1016/j.jneumeth.2003.10.009 15102499

[25] Tadel F Baillet S Mosher JC Pantazis D Leahy RM. Brainstorm: a user-friendly application for MEG/EEG analysis. Comput Intell Neurosci. (2011). 10.1155/2011/879716 21584256 PMC3090754

[26] Stam CJ Breakspear M van Walsum AMVC van Dijk BW. Nonlinear synchronization in EEG and whole-head MEG recordings of healthy subjects. Hum Brain Mapp. (2003) 19:63–78 10.1002/hbm.10106 12768531 PMC6872060

[27] Piotrowski T Rittweger J Zange JA. comparison of squatting exercise on a centrifuge and with earth gravity. Front Physiol. (2018) 9:1759. 10.3389/fphys.2018.01759 30568604 PMC6290078

[28] Rampello A Franceschini M Piepoli M Antenucci R Lenti G Olivieri D . Effect of aerobic training on walking capacity and maximal exercise tolerance in patients with multiple sclerosis: a randomized crossover-controlled study. Phys Ther. (2007) 87:545–55. 10.2522/ptj.20060085 17405806

[29] Rowell LB. Human Cardiovascular Control. New York: Oxford University Press. (1993).

[30] Ehsani AA Ogawa T Miller T R Spina RJ Jilka SM. Exercise training improves left ventricular systolic function in older men. Circulation. (1991) 83:96–103. 10.1161/01.CIR.83.1.96 1984902

[31] Artiles AD Heldt T Young LR. Effects of artificial gravity on the cardiovascular system: computational approach. Acta Astronaut. (2016) 126:395–410. 10.1016/j.actaastro.2016.05.005

[32] Clément GR Bukley AP Paloski WH. Artificial gravity as a countermeasure for mitigating physiological deconditioning during long-duration space missions. Front Syst Neurosci. (2015) 9:92. 10.3389/fnsys.2015.00092 26136665 PMC4470275

[33] Hiroyuki H Hamaoka T Sako T Nishio S Kime R Murakami M . Oxygenation in vastus lateralis and lateral head of gastrocnemius during treadmill walking and running in humans. Eur J Appl Physiol. (2002) 87:343–9. 10.1007/s00421-002-0644-y 12172872

[34] Bhambhani Y Maikala R Buckley S. Muscle oxygenation during incremental arm and leg exercise in men and women. Eur J Appl Physiol. (1998) 78:422–31. 10.1007/s004210050441 9809843

[35] Janssen AL Boster A Patterson BA Abduljalil A Prakash RS. Resting-state functional connectivity in multiple sclerosis: an examination of group differences and individual differences. Neuropsychologia. (2013) 51:2918–29. 10.1016/j.neuropsychologia.2013.08.010 23973635

[36] Sandroff BM Wylie GR Sutton BP Johnson CL DeLuca J Motl RW. Treadmill walking exercise training and brain function in multiple sclerosis: preliminary evidence setting the stage for a network-based approach to rehabilitation. Mult Scler J Exp Transl Clin. (2018) 4:205521731876064. 10.1177/2055217318760641 29497559 PMC5824908

[37] Zanchi D Cunningham G Lädermann A Ozturk M Hoffmeyer P Haller S. Brain activity in the right-frontal pole and lateral occipital cortex predicts successful post-operatory outcome after surgery for anterior glenoumeral instability. Sci Rep. (2017) 7:498 10.1038/s41598-017-00518-9 28356560 PMC5428665

[38] Akbar N Sandroff BM Wylie GR Strober LB Smith A Goverover Y Motl W DeLuca J Genova H. Progressive resistance exercise training and changes in resting-state functional connectivity of the caudate in persons with multiple sclerosis and severe fatigue: a proof-of-concept study. Neuropsychol Rehabil. (2020) 30:54–66. 10.1080/09602011.2018.1449758 29618280

[39] Tavazzi E Bergsland N Cattaneo D Gervasoni E Laganà MM Dipasquale O . Effects of motor rehabilitation on mobility and brain plasticity in multiple sclerosis: a structural and functional MRI study. J Neurol. (2018) 265:1393–401. 10.1007/s00415-018-8859-y 29627940

[40] Müller P Rehfeld K Schmicker M Hökelmann A Dordevic M Lessmann V . Evolution of neuroplasticity in response to physical activity in old age: the case for dancing. Front Aging Neurosci. (2017) 9:56. 10.3389/fnagi.2017.00056 28352225 PMC5348543

[41] Fyhn M Molden S Witter MP Moser EI Moser MB. Spatial representation in the entorhinal cortex. Science. (2004) 305:1258–64. 10.1126/science.1099901 15333832

[42] Stranahan AM Khalil D Gould E. Running induces widespread structural alterations in the hippocampus and entorhinal cortex. Hippocampus. (2007) 17:1017–22. 10.1002/hipo.20348 17636549 PMC2956984

[43] Peters J Dauvermann M Mette C Platen P Franke J Hinrichs T . Voxel-based morphometry reveals an association between aerobic capacity and grey matter density in the right anterior insula. Neuroscience. (2009) 163:1102–8. 10.1016/j.neuroscience.2009.07.030 19628025

[44] Williamson JW McColl R Mathews D Ginsburg M Mitchell JH. Activation of the insular cortex is affected by the intensity of exercise. J Appl Physiol. (1985) 1999:1213–9. 10.1152/jappl.1999.87.3.1213 10484598

[45] Colcombe SJ Erickson KI Scalf PE Kim JS Prakash R McAuley E . Aerobic exercise training increases brain volume in aging humans. J Gerontol A Biol Sci Med Sci. (2006) 61:1166–70. 10.1093/gerona/61.11.1166 17167157

[46] Zhang H Xu L Wang S Xie B Guo J Long Z . Behavioral improvements and brain functional alterations by motor imagery training. Brain Res. (2011) 1407:38–46. 10.1016/j.brainres.2011.06.038 21764038

[47] Cheng C Fan L Xia X Eickhoff SB Li H Li H Chen J Jiang T. Rostro-caudal organization of the human posterior superior temporal sulcus revealed by connectivity profiles. Hum Brain Mapp. (2018) 39:5112–25. 10.1002/hbm.24349 30273447 PMC6866551

[48] Steffener J Habeck C O'Shea D Razlighi Q Bherer L Stern Y. Differences between chronological and brain age are related to education and self-reported physical activity. Neurobiol Aging. (2016) 40:138–44. 10.1016/j.neurobiolaging.2016.01.014 26973113 PMC4792330

[49] Yu Q Herold F Becker B KluGah-Brown B Zhang Y Perrey S . Cognitive benefits of exercise interventions: an fMRI activation likelihood estimation meta-analysis. Brain Struct Funct. (2021) 226:601–19. 10.1007/s00429-021-02247-2 33675397

[50] Chen YS Chen HL Lu CH Chen MH Chou KH Tsai NW . Reduced lateral occipital gray matter volume is associated with physical frailty and cognitive impairment in Parkinson's disease. Eur Radiol. (2019) 29:2659–68. 10.1007/s00330-018-5855-7 30523452

[51] Benedict C Brooks SJ Kullberg J Nordenskjöld R Burgos J Le Grevès M . Association between physical activity and brain health in older adults. Neurobiol Aging. (2013) 34:83–90. 10.1016/j.neurobiolaging.2012.04.013 22592017

[52] Yang H Xu H Li Q Jin Y Jiang W Wang J . Study of brain morphology change in Alzheimer's disease and amnestic mild cognitive impairment compared with normal controls. Gen Psychiatr. (2019) 32:2. 10.1136/gpsych-2018-100005 31179429 PMC6551438

[53] Roosendaal SD Hulst HE Vrenken H Feenstra HE Castelijns JA Pouwels PJ . Structural and functional hippocampal changes in multiple sclerosis patients with intact memory function. Radiol. (2010) 255:595–604. 10.1148/radiol.10091433 20413769

[54] Buyukturkoglu K Porcaro C Cottone C Cancelli A Inglese M Tecchio F. Simple index of functional connectivity at rest in Multiple Sclerosis fatigue. Clin Neurophysiol. (2017) 128:807–13. 10.1016/j.clinph.2017.02.010 28340429

[55] https://vitalise-project.eu/.

